# Enhanced production of l-histidine in *Escherichia coli* through systematic metabolic engineering and CER controlled fermentation

**DOI:** 10.1016/j.synbio.2026.04.038

**Published:** 2026-05-25

**Authors:** Bo Zhang, Xintian Liu, Tianjun Zhao, Jinkang Hao, Zejian Wang, Menglei Xia, Ning Chen, Xiaonan Wang, Xiaoguang Fan

**Affiliations:** a College of Biotechnology, Tianjin University of Science & Technology, Tianjin, 300457, China; b State Key Laboratory of Bioreactor Engineering, East China University of Science and Technology, Shanghai, 200237, China

**Keywords:** l-histidine, *Escherichia coli*, Machine learning-guided transporter discovery, Non-model microbial mining, CER-Controlled fermentation

## Abstract

l-Histidine is an essential amino acid with important applications in pharmaceuticals and nutrition, highlighting the demand for efficient microbial production platforms. This study developed a high-performance *Escherichia coli* cell factory through systematic metabolic engineering. First, we mined a feedback-resistant *hisG∗*_smar_ and the entire mutant his operon from a previously obtained high l-histidine-producing mutant of *Serratia marcescens*, offering novel enzymatic parts beyond conventional sources. Combined with precursor supply enhancement and redox balancing, the engineered strain yielded 4.46 g/L l-histidine. Second, a machine learning-based platform (TransDW) was utilized to predict and validate a novel efflux transporter, Cgl1374, increasing titer to 4.82 g/L. Third, we implemented a growth phase-dependent system to dynamically regulate *pgi* expression, redirecting carbon flux and achieving 5.49 g/L in shake flasks. Finally, applying a novel carbon evolution rate (CER)-based control strategy in fed-batch fermentation, the optimized strain achieved 49.8 g/L of l-histidine in a 5-L bioreactor, with a yield of 0.265 g/g glucose, which is the highest yield reported for engineered *E. coli*. This work establishes a synergistic framework combining non-model gene discovery, computational transport engineering, and real-time physiological feedback control, providing a versatile blueprint for next-generation microbial cell factories.

## Highlights

• Comparative genomics-mined *Serratia marcescens his* operon resolving pathway bottlenecks.
• Engineered *pntAB/sthA* module balancing NADH/NADPH cofactor pools.
• Machine learning-predicted Cgl1374 transporter enhancing histidine efflux.
• Growth-phase-responsive *pgi* control optimizing carbon flux distribution.
• CER-controlled fermentation achieving 49.8 g/L titer and 0.265 g/g yield for l-histidine production.

## Graphical abstract

**Figure:**
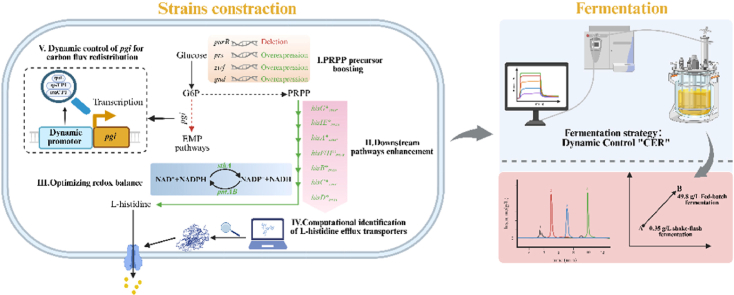

## 1 Introduction

**Fig. 1:**
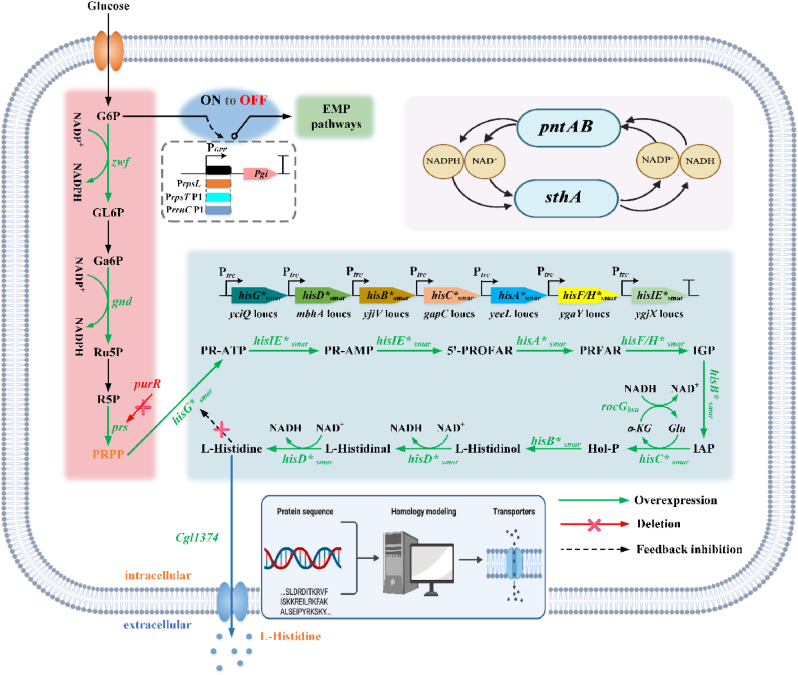
Biosynthetic pathway of l-histidine from glucose and the metabolic engineering strategies used in this study. Abbreviations: PRPP, phosphoribosyl pyrophosphate; G6P, glucose 6-phosphate; GL6P, gluconolactone-6-phosphate; Ga6P, glucosamine-6-phosphate; Ru5P, ribulose-5-phosphate; R5P, ribose-5-phosphate; PR-ATP, phosphoribosyl-ATP; PR-AMP, phosphoribosyl-AMP; 5′-ProFAR, phosphoribosylformimino-AICAR-phosphate; PRFAR, phosphoribulo-sylformimino-AICAR-phosphate; IGP, imidazole-glycerol phosphate; IAP, imidazole-acetol phosphate; Hol-P, L-histidinol phosphate; NAD^+^,oxidized nicotinamide adenine dinucleotide; NADH, reduced nicotinamide adenine dinucleotide; α-KG, α-ketoglutarate; Glu, glutamate. Blue arrows indicate overexpression of the relevant genes through chromosomal integration or promoter replacement; the red arrow has two diagonal lines on it to represent the deleted gene. Created with BioRender.com.

l-Histidine (His) is a biologically active and versatile amino acid with wide applications in health supplements, animal feed, and pharmaceuticals [1,2]. As an essential amino acid, l-histidine requires exogenous supplementation for physiological maintenance in both adults and infants [3]. Its derived metabolites, including ergothioneine [4], carnosine [5], and histamine [6], have been reported to mediate metabolic regulation, participate in immune responses, and serve as therapeutic agents for cancer and Parkinson's disease. Moreover, over 40% of approved monoclonal antibody (mAb) formulations include histidine or histidine hydrochloride to ensure stability [7]. Conventionally, manufacturing of l-histidine relied on chemical synthesis or protein hydrolysis, both of which present limitations [8,9]. Chemical synthesis generates racemic mixtures with environmental contamination risks [10], whereas protein hydrolysis is constrained by the limited availability of natural protein resources [8]. Microbial fermentation has thus emerged as a more sustainable, cost-effective alternative for large-scale production. The l-histidine biosynthetic pathway is ubiquitously distributed across diverse organisms including bacteria, fungi, and archaea [11]. Starting with phosphoribosyl pyrophosphate (PRPP) and adenosine triphosphate (ATP) as precursors, the pathway proceeds through ten enzymatic reactions catalyzed by nine enzymes encoded by the *his* operon (Fig. 1) [12]. The pathway is subject to dual regulation, where the end product binds to the rate-limiting enzyme HisG to exert feedback inhibition, and the regulatory peptide HisL controls operon expression through transcription attenuation [12,13]. Bioproduction of l-histidine has been investigated in various strains such as *Corynebacterium glutamicum*, *E. coli*, and *S*. *marcescens* [14]. Early strain improvement mainly relied on random mutagenesis and screening; however, many resulting mutants were auxotrophic with unclear genetic backgrounds, limiting their use in large-scale fermentation and precise engineering [15,16].

In recent years, advances in synthetic biology have enabled detailed elucidation and rational engineering of the l-histidine biosynthetic network [17]. Achieved an l-histidine titer of 66.5 g/L in *E*. *coli* through systematic metabolic engineering, including chromosomal pathway integration, enhanced PRPP and ATP supply, and export optimization [18]. Combined protein and metabolic engineering to construct a recombinant *C*. *glutamicum* strain that produced 5.07 g/L in a 3-L bioreactor. More recently, channel engineering and energy metabolism regulation were applied to optimize precursor supply, nitrogen utilization, and cofactor balance, achieving 52.32 g/L in 36 h with a glucose yield of 0.22 g/g in a 5-L fermenter [19]. These advances highlight the strong potential of microbial cell factories for high-efficiency l-histidine production. However, several challenges remain. First, most integrated *his* operons are derived from model organisms (e.g., *E. coli*, *C. glutamicum*) [20], leaving the rich biosynthetic potential of non-model or evolved industrial strains largely untapped.

For instance, a high l-histidine-producing mutant strain of *S. marcescens*, previously obtained in our laboratory through ARTP mutagenesis and screening, suggested the presence of unique and optimized pathway enzymes; however, its elevated broth viscosity and biosafety concerns rendered *S. marcescens* unsuitable for industrial-scale manufacturing. Therefore, the beneficial genetic traits need to be transferred into a more robust and industrially amenable chassis strain. Second, the identification of efficient product exporters remains a bottleneck. While broad-specificity transporters like LysE or ArgO have been used [21], dedicated or high-affinity l-histidine efflux transporters are unknown, and rational discovery methods are lacking. Third, current bioprocess control often relies on static parameters like dissolved oxygen (DO) or pH, which are indirect proxies of metabolic activity. A strategy directly linking real-time metabolic output (e.g., carbon evolution rate, CER) to nutrient feeding could better maintain metabolic homeostasis and prevent overflow metabolism.

To overcome current bottlenecks, this study performed whole-genome resequencing of the *S. marcescens* mutant CGMCC 19116, identified key *his* operon mutations, and introduced them into *E. coli* MG1655 to relieve feedback inhibition and enhance pathway flux. Precursor supply was optimized by engineering the PRPP synthesis module, and a glutamate regeneration cycle combined with a transhydrogenase system was implemented to balance redox status. The TransDW platform was used to identify transporters, while growth phase-dependent promoters enabled dynamic redistribution of carbon flux. Finally, a CER-based fermentation strategy was established to coordinate metabolic activity with nutrient feeding and minimize overflow metabolism. This work not only constructs an efficient l-histidine producer but also establishes a comprehensive methodological framework that integrates genomics, predictive computational tools, synthetic biology, and advanced bioprocess engineering for developing microbial cell factories.

## 2 Materials and methods

### 2.1 Strains, growth medium and reagents

All bacterial strains and plasmids used in this study are listed in Supplementary Materials. *E. coli* MG1655 served as the parental strain for constructing the l-histidine cell factory. *E. coli* DH5α was used for plasmid construction, and *C*. *glutamicum* ATCC 13032 and *Bacillus subtilis* 168 were used as gene sources.

*E. coli* cells were cultured in Luria–Bertani (LB) medium supplemented with spectinomycin (100 μg/mL) or ampicillin (100 μg/mL) when necessary. Recombinant strains were cultured at 32, 37 or 42 °C as appropriate. Isopropyl-β-d-1-thiogalactopyranoside (IPTG) and l-arabinose were added as inducers at concentrations of 0.2 mmol/L and 2 g/L, respectively.

The seed medium comprised 25 g/L glucose, 5 g/L yeast extract, 1 g/L peptone, 1 g/L citric acid, 1.8 g/L K_2_HPO_4_·3H_2_O, 0.5 g/L MgSO_4_·7H_2_O, 10 mg/L MnSO_4_·H_2_O, 10 mg/L FeSO_4_·7H_2_O, and 1 mg/L Vitamin B Mix (pH 6.5−7.5). The fermentation medium comprised 20 g/L glucose, 4 g/L yeast extract, 2 g/L Citric acid, 3.5 g/L K_2_HPO_4_·3H_2_O, 1.8 g/L MgSO_4_·7H_2_O, 0.2 g/L methionine, 10 mg/L MnSO_4_·H_2_O, 20 mg/L FeSO_4_·7H_2_O, 1 g/L sodium glutamate and 2 mg/L Vitamin B Mix (pH 6.5−7.5). Vitamin B Mix contains 1 g/L VB_1_, 1 g/L VB_3_, 1 g/L VB_5_ and 1 g/L VB_12_. Phenol red (8 mg/L) was added during shake flask fermentation.

PrimeSTAR HS DNA polymerase, polymerase chain reaction (PCR) reagents, and DNA purification kits were obtained from Takara (TaKaRa, Beijing, China). Yeast extract and peptone were purchased from Lesaffre (Shanghai, China). The inorganic salts were purchased from Sinopharm Chemical Reagent Co., Ltd. (Shanghai, China). The antibiotics and amino acids were purchased from Aladdin (Shanghai, China)

### 2.2 CRISPR/Cas9 genome editing system

Supplementary Materials present the primers used in this study. Gene deletion and integration were performed using the CRISPR/Cas9-based gene editing system [22]. The plasmids pREDCas9 and pGRB used for CRISPR/Cas9-mediated gene editing system were kindly provided by Prof. Tao Chen in Tianjin University [23].

The integration of *gnd* with the pseudogene *rph* locus was used as a reference, and donor DNA containing the homologous arms of *yciQ* and P_*trc*_-*hisG*∗_smar_-T_*trc*_ was obtained through overlapping PCR. *E. coli* MG655 was used as the template, and the primer pairs, *yciQ*−U–S/*yciQ*-U-A, *hisG*∗_smar_-S/*hisG*∗_smar_-A, and *yciQ*-D-S/*yciQ*-D-A were employed. To construct plasmid pGRB-*yciQ*, the sequence targeting *yciQ* was annealed to form double-stranded DNA using P- *yciQ*-S and P- *yciQ*-A. The linearized pGRB vector was obtained via PCR using primers pGRB-S and pGRB-A. Then, sequence targeting *yciQ* was connected with linearized pGRB vector through homologous recombination. The pGRB-*yciQ* plasmid and the donor DNA were co-transformed into competent ZL4. After cultivation in LB medium for 1 h, the cells were plated on LB agar with 100 μg/mL ampicillin and 50 μg/mL spectinomycin. Positive colonies were selected using colony PCR and identified using DNA sequencing.

### 2.3 Whole genome sequencing

The genomic DNA of *S*. *marcescens* CGMCC No. 19116 was extracted using the GenElute Bacteria DNA Kit (Sigma-Aldrich, Germany) following the manufacturer's protocols. The genomic DNA from *S*. *marcescens* CGMCC No. 19116 was sequenced using the RS II platform (PACB, Pacific Biosciences, American) and NovaSeq 6000 (Illumina, American) at the Guangzhou Kidio Biotechnology Ltd (Guangzhou, China). The third-generation sequencing reads were assembled using Falcon software. The second-generation sequencing reads were aligned to assembled genome sequences using Pilon (version 1.23), and the genome results were corrected according to the software default parameters. The genomes of the wild-type strain *S. marcescens* SM39 and the previously obtained high l-histidine-producing mutant strain *S. marcescens* CGMCC No.19116 were compared using LASTZ (http://www.bx.psu.edu/miller_lab/dist/README.lastz-1.02.00/) and MUMMer software (http://mummer.sourceforge.net/) [24]. *S. marcescens* SM39 complete genome as a reference sequence (NCBI Reference Sequence: AP013063.1).

### 2.4 Transporter prediction

Proteome datasets of *E. coli* MG1655 and *C. glutamicum* ATCC13032 were downloaded from NCBI. Protein sequences were processed using in-house Python scripts to generate CSV prediction files. l-histidine substrate information was encoded in InChI format. The TransDW web server (https://www.mtc-lab.cn/TransDW) was used to predict transporters via three pretrained models: i) UTP model (identifies all transporters) ii) DirectIO model (predicts the direction of substrate transporters) iii) SPOTIC model (predicts the possibility of transporters binding to specific substrates). After the calculation is completed, the server will generate a comprehensive forecast report and send it to the user's browser.

### 2.5 Shake-flask fermentation

l-histidine-producing strains were inoculated into 500-mL shake flasks containing 30 mL seed medium and were cultivated at 37 °C (180 rpm) until the OD_600_ reached approximately 10. The seed cultures (3 mL) were transferred to a 500-mL baffled shake flask containing 30 mL of fermentation medium and cultivated at 37 °C and 180 rpm for 28 h. During fermentation, the pH was maintained at 6.5 ± 0.2 using ammonia (25% v/v) (based on the change in the color of the medium), and 1 mL of glucose solution (60% w/v) was added when glucose was depleted.

### 2.6 Fed-batch fermentation in a 5-L bioreactor

Recombination strains cultured on agar slants were transferred to a 5-L bioreactor (Parallel Bioreactor System, Shanghai, China) containing 2.5 L of fermentation medium and cultivated at 37 °C until the OD_600_ reached 15–20. Subsequently, 400 mL of the seed culture was inoculated into another 5-L bioreactor containing 2.5 L of fermentation medium. The temperature and dissolved oxygen were maintained at 34 °C and 15–45%, respectively. During the whole fermentation process, the pH value (7.1–7.3) was automatically maintained using ammonia. The sterile glucose solution (80%, w/v) was automatically supplied when the glucose was exhausted in the initial medium.

### 2.7 Analytical methods

Cell density was measured at 600 nm (OD_600_) with a UV spectrophotometer. Culture supernatants were centrifuged, and l-histidine was quantified using a Hitachi L-8900 amino acid analyzer (Hitachi, Japan) with a 4.6 × 60 mm column (3 μm resin). Flow rate: 0.4 mL/min; derivatization temperature: 135 °C; detection wavelengths: 570 nm and 440 nm.

### 2.8 Statistical analysis

All experiments were performed in triplicate, and data are reported as mean ± standard deviation (SD). Statistical significance was evaluated using two-tailed Student's t-test with *p* < 0.05 in GraphPad Prism 8.3.0.

## 3 Results and discussion

### 3.1 Identification of his operon mutations in a high l-histidine-producing mutant of *S. marcescens*

In recent years, advances in synthetic biology have enabled integrated approaches that combine mutagenesis, high-throughput screening, and comparative genomics, offering effective strategies for optimizing microbial cell factories [25]. Comparative genomics, in particular, enables genome-wide comparisons between wild-type and engineered strains to identify mutations underlying desirable phenotypes, guiding rational metabolic engineering efforts. In our earlier work, a high l-histidine-producing mutant strain, *S. marcescens* CGMCC No.19116, was obtained in our laboratory through ARTP mutagenesis and screening of the wild-type strain *S. marcescens* SM39 and was preserved as a laboratory stock strain. In the present study, this mutant was used for whole-genome resequencing and comparative genomic analysis. CGMCC No.19116 produced 20.3 g/L l-histidine in a 5-L bioreactor.

To uncover the genetic basis for this phenotype, we performed hybrid genome sequencing using PacBio (third-generation) and Illumina (second-generation) platforms and the key genomic features are summarized in Table 1. Comparison with the reference genome of *S. marcescens* SM39 revealed multiple mutations in the *his* operon closely linked to l-histidine biosynthesis. Specifically, nonsynonymous substitutions and one insertion were identified across all nine genes of the operon (*hisG*, *hisD*, *hisB*, *hisC*, *hisA*, *hisF/H*, and *hisIE*), suggesting altered enzymatic activity that may contribute to the strain's high-yield phenotype (Table 2). These results identify validated genetic elements that can support rational pathway engineering in *E. coli*–based l-histidine production strains.

**Table 1:** Annotation information of *S. marcescens* CGMCC No. 19116 strain.

Item	Quantity
Gene size (bp)	5068351
GC content (%)	59.7
Geng coding	4642
Geng island	4
Total length of code area (bp)	4402950
Total length of code gene island (bp)	50243
Tandem repeat	107
rRNA	22
tRNA	90
sRNA	34
SINEs	30
LINEs	33
Prophage	2
Total prophage length (bp)	53593

**Table 2:** Comparison of l-histidine operon gene mutations.

Characteristics	Number	mutation types	PCR confirmation
*hisG*∗_smar_	3	Replacement:A10T; E19D; C149Y	Yes
*hisD*∗_smar_	10	Replacement:S38 N; K45E; D63,91E; A65S; A66T; C78A; E284,297,338D	Yes
*hisB*∗_smar_	2	Replacement:N121D; A122T	Yes
*hisC*∗_smar_	5	Replacement:G69E; M141L; N167S; L171 M; K175R	Yes
*hisA*∗_smar_	2	Replacement:K12 N; R86Q	Yes
*hisF/H*∗_smar_	8	Replacement:K84,326R; E111,118D; I154V; L195R; A199V; V352I	Yes
*hisIE*∗_smar_	7	Replacement:N7R; A44T; V193A; Insert: 203-206QKKA	Yes

### 3.2 Modification of the core biosynthetic pathway for l-histidine production in *E. coli*

#### 3.2.1 Screening of HisG variants for improved l-histidine production

In *E*. *coli,* ATP phosphoribosyltransferase (HisG) catalyzes the first step of l-histidine biosynthesis and is subject to feedback inhibition by l-histidine [26]. To alleviate this rate-limiting constraint, engineered HisG mutants have been developed to reduce sensitivity to l-histidine binding [27]. Previous systematic evaluation of five HisG variants from *E. coli* (HisG_eco_) or *C. glutamicum* (HisG_cgl_) identified HisG_cgl_^S143F^ as enabling maximal l-histidine production [17]. This study screened three HisG enzymes: the wild-type HisG_smar_ from *S*. *marcescens*, the mutant HisG∗_smar_ from *S. marcescens* CGMCC No.19116, and the *C. glutamicum* HisG_cgl_^S143F^. Genes encoding these enzymes, controlled by the trc promoter, were individually integrated into the *yciQ* chromosomal locus of *E. coli* ZL1, generating recombinant strains ZL1-1, ZL1-2, and ZL1-3, respectively.

**Fig. 2:**
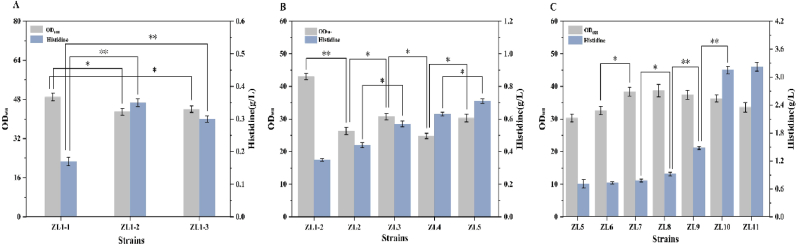
Stepwise optimization of the l-histidine pathway in *E. coli*. Effects on l-histidine titer and biomass (OD_600_). (A) Feedback relief via integrated *hisG* variants (P_*trc*_) at *yciQ*: wild-type *hisG* from *S. marcescens* (ZL1-1), mutant *hisG∗*_smar_ (ZL1-2), and *hisG*_cgl_^S143F^ (ZL1-3). l-histidine titer and final OD_600_ after shake-flask fermentation. (B) PRPP supply strengthening: Δ*purR* (ZL2), *prs* overexpression (ZL3), and further reinforcement of the pentose phosphate pathway through overexpression of *zwf* (ZL4) and *gnd* (ZL5). (C) Downstream operon enhancement: sequential P_*trc*_-driven integration of mutant *S. marcescens* genes to generate ZL6–ZL11; Data shown are mean ± SD (n = 3 independent experiments), ∗ indicates p < 0.05, ∗∗ indicates p < 0.01.

As shown in Fig. 2A, overexpression of these *hisG* variants significantly affected both l-histidine production and cell growth. Strain ZL1-2, expressing *hisG*∗_smar_, produced 0.35 g/L l-histidine, which represents a 105% and 16.7% increase compared to ZL1-1 and ZL1-3, respectively. Furthermore, while strain ZL1-1 harboring wild-type *hisG*_*smar*_ exhibited the highest final OD_600_, it yielded the lowest l-histidine production. To further evaluate whether the superior performance of *hisG∗*_*smar*_ was associated with reduced sensitivity to feedback pressure at the cellular level, exogenous l-histidine supplementation experiments were performed. When increasing concentrations of l-histidine (0–5 mM) were added to the fermentation medium, all three strains exhibited decreased l-histidine production. However, strain ZL1-2 consistently maintained higher l-histidine titers than ZL1-1 and ZL1-3 under increasing extracellular histidine concentrations (Table S5). In particular, ZL1-2 retained a larger fraction of its production capacity under feedback pressure, whereas ZL1-1 showed the most pronounced decline. Furthermore, to gain preliminary structural insight into the improved performance of *hisG∗*_*smar*_, we compared the predicted structures of HisG_smar_ and HisG∗_smar_ based on the reported crystal structure of ATP phosphoribosyltransferase from *Mycobacterium tuberculosis* (*Mt*ATP-PRT; PDB ID: 1NH7) [28], in which the residues involved in l-histidine-mediated allosteric inhibition have been experimentally characterized. The analysis suggested that several putative residues (Glu246, Thr252, and Ala286) involved in histidine recognition were positioned farther from the putative histidine-binding region in HisG∗_smar_ than in HisG_smar_, which may partially explain its reduced sensitivity to l-histidine-mediated feedback inhibition (Fig. S2). These results indicate that *hisG∗*_*smar*_ is a superior HisG variant for improving l-histidine production in the engineered *E. coli* background used in this study.

#### 3.2.2 PRPP precursor boosting

In *E. coli*, l-histidine biosynthesis initiates with the HisG-catalyzed transfer of a phosphoribosyl group from PRPP to the adenine ring of ATP, forming phosphoribosyl-ATP (PR-ATP) [29]. PRPP serves as the exclusive donor of the l-histidine carbon skeleton and is a key substrate triggering the synthesis pathway [18]. Consequently, enhancing PRPP supply through strategies such as optimizing *prs* expression [30], deregulating feedback inhibition [31], or strengthening the pentose phosphate pathway (PPP) [23], constitutes a core approach in metabolic engineering for improving l-histidine production.

PRPP biosynthesis primarily relies on PRPP synthetase (encoded by the *prs* gene), which catalyzes the condensation of ribose-5-phosphate (R5P) and ATP to generate PRPP [32]. However, *prs* gene expression is transcriptionally repressed by the PurR protein, limiting PRPP synthesis. To address this limitation, this study first knocked out the *purR* gene i*n E. coli* ZL1-2, yielding strain ZL2. Subsequently, the *prs* gene under the control of the P_*trc*_ promoter was integrated into the *yghx* locus to construct strain ZL3. Shake-flask fermentation results (Fig. 2B) showed that compared to ZL1-2, ZL2 exhibited a 25.7% increase in l-histidine titer to 0.44 g/L, but its OD_600_ decreased by 38.8%. ZL3 achieved a further increase in l-histidine titer to 0.57 g/L. The impaired cell growth was likely due to excessive consumption of R5P and ATP resulting from enhanced PRPP synthesis, leading to an imbalance in metabolic resource allocation. To ensure adequate R5P supply and alleviate the carbon flux bottleneck in its synthesis pathway, we sequentially overexpressed the *zwf* and *gnd* genes in ZL3, generating strains ZL4 and ZL5. The results (Fig. 2B) demonstrated that ZL4 achieved a l-histidine titer (0.63 g/L) and biomass 10.5% and 19.2% higher than ZL3, respectively. ZL5 further increased l-histidine titer (0.71 g/L) and biomass by 12.7% and 22.1%, respectively. This demonstrates that strengthening PPP synergistically improves both l-histidine production and cell growth by boosting PRPP supply.

#### 3.2.3 Downstream pathways enhancement

With precursor supply optimized, we hypothesized that inefficient downstream pathway conversion would become the limiting factor. Therefore, To address this, seven *his* operon genes from operon genes (*hisD∗*_*smar*_, *hisB∗*_*smar*_, *hisC∗*_*smar*_, *hisA∗*_*smar*_, *hisF/H∗*_*smar*_, and *hisIE∗*_*smar*_) from *S*. *marcescens* CGMCC No.19116, each under P_*trc*_ control, were sequentially integrated into *E. coli* ZL5 at designated loci, generating strains ZL6–ZL11. As shown in Fig. 2C, strain ZL11 expressing all seven operon genes produced 3.22 g/L l-histidine, representing a 4.53-fold titer and 10.8% biomass increase over ZL5, confirming the benefit of downstream pathway enhancement. Notably, stagewise integration revealed distinct contributions: ZL8, ZL9, and ZL10 successively reached 0.92, 1.48, and 3.15 g/L in titer, with modest biomass changes, demonstrating cumulative benefits of *hisC*∗, *hisA*∗, and *hisF/H*∗ expression. The integration of *hisC∗*_*smar*_, *hisA∗*_*smar*_, and *hisF/H∗*_*smar*_ collectively contributed 82.7% of the total titer increment, suggesting that overexpressing these heterologous enzymes alleviates critical pathway bottlenecks: histidinol phosphate transamination (catalyzed by HisC), imidazole glycerol-phosphate synthesis (HisA), and ATP-dependent cyclization (HisF/H).

### 3.3 Glutamate precursor regeneration and cofactor balancing

**Fig. 3:**
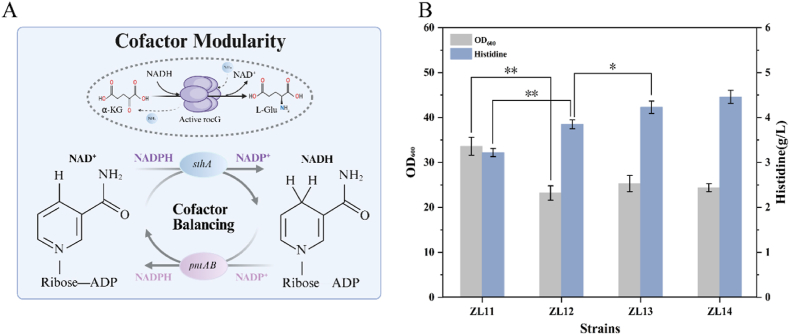
Modification of the transhydrogenase system and validation of NADH/NADPH redox balance regulation. (A) Engineering the transhydrogenase system to establish an intracellular NADH/NADPH redox cycle. (B) Effects of equilibrium of glutamate regeneration and redox flux on histidine production and cell growth. Data shown are mean ± SD (n = 3 independent experiments), ∗ indicates p < 0.05, ∗∗ indicates p < 0.01. Created with BioRender.com.

Even with optimization of the core l-histidine biosynthetic pathway, production remained limited by additional bottlenecks. A major constraint was insufficient l-glutamate for the HisC-catalyzed transamination step. In this step, l-glutamate donates its α-amino group to IAP, producing L-histidinol phosphate (HOL-P) and α-ketoglutarate (α-KG) [12,17]. To recycle l-glutamate, the *rocG* gene from *B*. *subtilis* was chromosomally integrated under P_*trc*_ promoter control (Fig. 3A). This enzyme catalyzes NADH-dependent reductive amination of α-KG to regenerate l-glutamate. As shown in Fig. 3B, strain ZL12 carrying *rocG* exhibited a 19.5% increase in l-histidine titer (3.85 g/L), consistent with the findings of Wu et al. However, biomass decreased by 30.9%, suggesting that redox imbalance likely disrupted metabolic flux and growth.

Redox cofactor imbalance is one of the major bottlenecks in biosynthesis, and balancing NADH/NADPH availability is critical to alleviate metabolic stress and meet biosynthetic demands [33,34]. In l-histidine synthesis, HisD catalyzes the final two oxidation steps, consuming NAD^+^ and generating NADH [35]. Besides, as shown in Section 3.2.2, enhancing the PPP significantly improved both cell growth and l-histidine production. This effect is likely attributable to increased NADPH supply, as the PPP is the primary source of NADPH [36]. Considering the dual requirement for NADPH and NAD^+^ in l-histidine biosynthesis, the P_*trc*_-driven *pntAB* gene was integrated into ZL12 to construct strain ZL13. PntAB catalyzes the unidirectional conversion of NADH to NADPH while promoting NAD ^+^ regeneration, supporting HisD activity [37]. This strategy increased l-histidine production by 9.9% to 4.23 g/L, though persistent NADH consumption may lead to reduced cellular reducing power.

To further optimize redox cofactor system, the P_*trc*_-driven *sthA* gene encoding the soluble bidirectional transhydrogenase SthA was introduced to complement PntAB, resulting in ZL14 [38]. Co-expression of these directionally complementary transhydrogenases established a dynamic system capable of responding to cellular redox demands: SthA provides NADH during high-demand phases (e.g., active glycolysis), while PntAB ensures a steady NADPH supply. This strategy increased the l-histidine titer to 4.46 g/L, representing a 5.4% improvement over ZL13 (PntAB only) and a 12.8% improvement over ZL12. Overall, coordinated expression of *rocG* and *pntAB/sthA* enabled efficient regeneration of the amino donor l-glutamate and maintained redox balance, thereby alleviating metabolic stress caused by redox imbalance.

### 3.4 Computational identification and validation of l-histidine efflux transporters

**Fig. 4:**
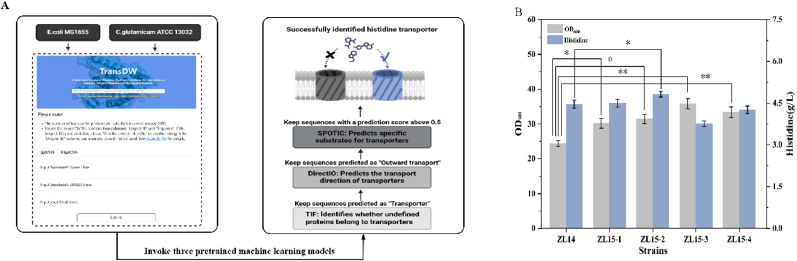
Computational platform–based simulations for the identification of l-histidine efflux transporters. (A) Schematic diagram of the strategy of screening l-histidine transporter in simulation prediction. (B) Effects of distinct transporters harbored by strains ZL15-1, ZL15-2, ZL15-3, and ZL15-4 on l-histidine production and cellular growth. Data shown are mean ± SD (n = 3 independent experiments), ∗ indicates p < 0.05, ∗∗ indicates p < 0.01.

Enhanced product excretion via transporter engineering serves as a critical strategy to optimize microbial cell factories by addressing intracellular toxicity accumulation, feedback inhibition, and transmembrane transport bottlenecks, thereby establishing robust driving forces for continuous biosynthesis [39]. Previous studies confirm that heterologous transporters improve l-histidine efflux efficiency, exemplified by *C*. *glutamicum* LysE expression in *E. coli* increasing titers 16.8% despite high Km limitations, and EcArgO overexpression enhancing yields by 10.7% [17]. However, dedicated l-histidine efflux transporters remain uncharacterized. In this study, we implemented the TransDW prediction platform where computational workflows sequentially deploy three machine learning models: TIF evaluates candidate proteins to generate prediction scores (retaining candidates >0.5 in transporter sets, with higher scores indicating greater target likelihood); DirectIO predicts transport directionality; and SPOTIC identifies substrate specificities (Fig. 4A). This pipeline identified three efflux-competent transporters after screening for outward transport directionality and >0.5 l-histidine transport probability: *Cgl2458* and *Cgl1374* from *C. glutamicum*, and *leuE* from *E. coli*. These genes were integrated into *yghE* locus of strain ZL14 under P_*trc*_ control, respectively, generating strains ZL15-1 (*Cgl2458*), ZL15-2 (*Cgl1374*), and ZL15-3 (*leuE*). As shown in Fig. 4B Shake-flask fermentation revealed divergent phenotypes: ZL15-1 restored cell growth without titer changes; ZL15-2 increased l-histidine titer 8% to 4.82 g/L with 77.7% biomass enhancement; ZL15-3 reduced titer 15.7% to 3.76 g/L despite 112% OD_600_ increase. We attribute the growth recovery in ZL15-1 and ZL15-2 to l-histidine efflux alleviating feedback inhibition. Notably, ZL15-2 expressing *Cgl1374* achieved superior l-histidine titer (4.82 g/L), demonstrating potent threshold-insensitive efflux capacity. Furthermore, strain ZL15-4 expressing *C. glutamicum lysE* under P_*trc*_ promoter control at the *yghE* locus showed no significant improvement in l-histidine titer. This result corroborates well-established literature demonstrating limited efficacy of transporter engineering below product threshold concentrations.

To evaluate the efflux capability of the selected transporter Cgl1374, intracellular and extracellular l-histidine concentrations were quantified in the control strain ZL14 and the transporter-expressing strain ZL15-2 under the same shake-flask fermentation conditions. As shown in Fig. S3, ZL15-2 exhibited a higher extracellular l-histidine concentration than ZL14, while its intracellular l-histidine level was lower (29.13 ± 4.20 mg/g DCW) than that of ZL14 (42.44 ± 3.41 mg/g DCW). These results show that Cgl1374 facilitated l-histidine export and reduced intracellular histidine accumulation.

### 3.5 Dynamic control of *pgi* for carbon flux redistribution

**Fig. 5:**
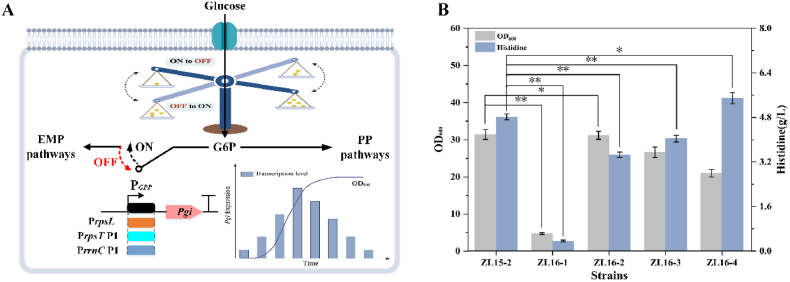
Redirecting carbon flux allocation at key metabolic nodes. (A) Growth-phase-dependent promoters replace the native pgi promoter to maintain high expression during exponential growth and attenuated expression in stationary phase, redirecting flux toward the PPP and histidine synthesis. Promoters: rpsL (weak), rpsT P1 (medium), rrnC P1 (strong). (B) Effects of implementing dynamic regulation strategies on l-histidine production and cell growth: *pgi* knockout (ZL16-1); GPP-regulated strains (ZL16-2/-3/-4). Data shown are mean ± SD (n = 3 independent experiments), ∗ indicates p < 0.05, ∗∗ indicates p < 0.01. Created with BioRender.com.

Minimizing carbon loss is a bottleneck in optimizing l-histidine-producing cell factories. Redirecting flux between the pentose phosphate and glycolytic pathways has proven effective for improving carbon utilization [40]. However, static regulation approaches, such as knockout or attenuation of *pgi* expression, often compromise growth and disrupt metabolic balance. We reasoned that dynamic control of *pgi*, which could adjust its expression according to physiological needs, would be a superior strategy [24]. To test this, we first constructed a negative control strain, ZL16-1, by deleting *pgi* in the parental strain ZL15-2. As shown in Fig. 5B, ZL16-1 exhibited severe impairments in both growth (79% decrease in OD_600_) and l-histidine production (92.5% reduction in titer), confirming the necessity of fine-tuned rather than complete blockade of this node.

To implement dynamic control, we designed an auto-switching system based on growth phase-dependent promoters (GPP). The design principle was to maintain high pgi expression during exponential growth to sustain glycolytic flux for biomass accumulation, and then reduce its expression in the stationary phase to redirect carbon toward l-histidine synthesis (Fig. 5A). This approach is supported by prior successful applications of analogous GPP systems for dynamic metabolic regulation. For instance, a similar promoter was shown to drive growth phase-dependent expression of *sucA*, supporting TCA cycle flux during growth and then redirecting carbon toward biosynthesis upon entry into stationary phase [24]. Leveraging this established principle, we extended its application to the critical glycolytic/PPP node by using GPPs to regulate *pgi*.

Based on this design, we selected three GPP promoters representing weak (rpsL), medium (rpsT P1), and strong (rrnC P1) activity levels (see Supplementary Materials) [41,42]. These promoters replaced the native *pgi* promoter in strain ZL15-2, generating strains ZL16-2, ZL16-3, and ZL16-4. All engineered strains exhibited improved growth compared with the negative control ZL16-1 (Fig. 5B). Notably, ZL16-4, which carries the strong rrnC P1 promoter, delivered the best performance: it increased l-histidine titer by 13.9% (from 4.82 to 5.49 g L^−1^) relative to ZL15-2, despite a 33.1% reduction in biomass. This improvement underscores the effectiveness of the dynamic-control strategy, which maintains sufficient metabolic flux for biomass formation during growth while redirecting carbon toward product synthesis in the stationary phase. Such temporal regulation alleviates growth inhibition and enhances production.

### 3.6 Fed-batch fermentation optimization via carbon evolution rate control

**Fig. 6:**
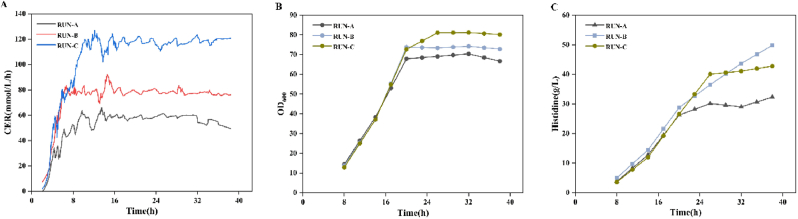
Fed-batch fermentation of ZL16-4 under carbon dioxide evolution rate (CER) control in a 5-L bioreactor. (A) Use CER dynamic indicator to monitor CER levels during fermentation. (B) OD_600_ time courses at different CER setpoints. (C) l-histidine titer time courses at different CER setpoints.

To evaluate the industrial production potential of the engineered strain ZL16-4, aerobic fed-batch fermentation was carried out in a 5-L bioreactor. Off-gas analysis using a process mass spectrometer (SHP8400PMS) provided real-time monitoring of physiological parameters, serving as direct indicators of cellular metabolic activity. Among these parameters, the CER reflects respiratory activity and substrate utilization efficiency, offering real-time insights into metabolic flux dynamics [43]. However, using CER as a control parameter has rarely been explored. We hypothesized that dynamically adjusting the glucose feed (800 g/L) and aeration rates based on real-time CER could maintain metabolic homeostasis and improve yield. During fed-batch fermentation, glucose feeding, agitation speed, and aeration rate were dynamically adjusted according to the CER signal to maintain the desired metabolic state. The corresponding time-course profiles of oxygen uptake rate (OUR), glucose feeding rate, agitation speed, and aeration rate under different CER setpoints are shown in Supplementary Fig. S4. Accordingly, the CER was precisely controlled at three setpoints: 60 ± 3.8, 80 ± 2.5, and 120 ± 6.9 mmol/L/h (Fig. 6).

As shown in Fig. 6B, the specific growth rate increased with CER, confirming a positive correlation between CER and metabolic activity. However, l-histidine production did not follow this trend (Fig. 6C). Although the highest CER (120 mmol/L/h) supported the fastest growth, it resulted in a lower final l-histidine titer (42.8 g/L) compared to that at 80 mmol/L/h (49.8 g/L). This suggests that at very high CER, an excessive proportion of carbon flux was diverted toward respiration and biomass formation, at the expense of product synthesis. Notably, acetate accumulation decreased substantially as CER increased (Table S4), indicating improved suppression of overflow metabolism under higher metabolic throughput. Crucially, the intermediate CER setpoint of 80 mmol/L/h struck an optimal balance: it maintained a high specific growth rate (0.606 h^−1^), minimized acetate accumulation to a low level (0.5 g/L), and maximized both l-histidine titer (49.8 g/L) and yield (0.265 g/g glucose). This study demonstrates that CER can be effectively employed as an independent, real-time control parameter to coordinate nutrient feeding with metabolic demand during scale-up.

### 3.7 Comparative analysis and strategic innovation

The pursuit of high-level l-histidine production in *E. coli* has seen significant achievements, most notably the 66.5 g/L titer reported by Wu et al. (2020) through systematic modular engineering [17]. More recently, Yang et al. (2025) achieved 52.32 g/L by innovatively applying channel engineering (Cip scaffolds) to precursor pathways [19]. When positioning our own results (49.8 g L^−1^, 0.265 g g^−1^ glucose) within this context, it becomes necessary to broaden the evaluation criteria beyond titer alone and to place greater emphasis on strategic innovation and methodological integration.

Table 3 provides a systematic comparison of the key features of this study with those of representative previous works. This comparison underscores several distinct contributions of our approach. First, we provide a genomics-driven pipeline for extracting valuable genetic parts from non-model, industrially relevant mutants, expanding the synthetic biology toolkit. Second, we demonstrate a computational-experimental workflow (TransDW) for discovering novel transporters, a generic approach applicable to other metabolites. Third, the CER (carbon evolution rate)-based control strategy developed and validated in this study represents a paradigm shift in process control: from static regulation based on environmental parameters (e.g., dissolved oxygen, pH) toward dynamic, physiology-guided bioprocessing that responds to real-time cellular metabolic activity. By directly coupling nutrient feeding with core metabolic fluxes, this strategy effectively maintains metabolic homeostasis.

**Table 3:** Comparative analysis of key metrics in high-yield l-histidine production by engineered *E. coli*.

Aspect	Wu et al. (2020) [17]	Yang et al. (2025) [19]	This Work
Core HisG Source	*C. glutamicum* (model)	*C. glutamicum* (model)	ARTP-evolved *S. marcescens* (non-model)
Key Engineering Focus	Modular pathway optimization, Purine coupling	Channel engineering (Cip) for precursors	Multi-strategy integration: Non-model genes, ML transport, Dynamic control
Transporter Strategy	Heterologous LysE (known)	Heterologous EcArgO (known)	De novo prediction & validation of Cgl1374 (ML-guided)
Process Control	Conventional DO/pH	Conventional DO/pH	CER-based real-time feedback control
Performance	66.5 g/L, 0.23 g/g	52.32 g/L, 0.22 g/g	49.8 g/L, 0.265 g/g

The highest glucose-to-product yield (0.265 g g^−1^) achieved here is direct evidence of the success of this integrated strategy in optimizing carbon economy. Although the absolute titer obtained in the MG1655 chassis is not the highest reported, the synergistic combination of multiple novel strategies presented in this work provides a more versatile research framework.

## 4 Conclusion

This study demonstrates an integrated strategy for efficient l-histidine production by combining comparative genomics, metabolic engineering, and process optimization. Key mutations from *S. marcescens* were introduced into *E. coli* to relieve feedback inhibition, while precursor supply, downstream flux, and redox balance were systematically optimized. Machine learning identified the transporter Cgl1374 to enhance product efflux, and growth phase-responsive *pgi* regulation improved carbon allocation. A CER-guided fermentation strategy minimized overflow metabolism, achieving 49.8 g/L l-histidine with a yield of 0.265 g/g glucose in a 5-L bioreactor. This work offers practical metabolic engineering strategies and scalable approaches for building microbial cell factories, paving the way for industrial production of l-histidine.

## CRediT authorship contribution statement

**Bo Zhang:** Writing – original draft, Visualization, Validation, Methodology, Investigation, Formal analysis, Data curation. **Xintian Liu:** Methodology, Investigation. **Tianjun Zhao:** Validation, Investigation. **Jinkang Hao:** Visualization, Software. **Zejian Wang:** Validation. **Menglei Xia:** Software. **Ning Chen:** Conceptualization. **Xiaonan Wang:** Writing – review & editing, Project administration, Data curation. **Xiaoguang Fan:** Supervision, Resources, Funding acquisition, Conceptualization.

## Funding

This work was supported by the Key Technologies Research and Development Program of China (Grant No. 2022YFD2101401) and the Special Fund Project for Science and Technology Innovation Strategy of Guangdong Province, China (Grant No. STKJ2023051).

## Declaration of competing interest

The authors declare that they have no known competing financial interests or personal relationships that could have appeared to influence the work reported in this paper.

## Abbreviations

PRPP, Phosphoribosyl Pyrophosphate; ATP, Adenosine Triphosphate ATP; CER, Carbon-dioxide Evolution Rate; OUR, Oxygen Uptake Rate; LB, Luria–Bertani; IPTG, Isopropyl-β-d-1-thiogalactopyranoside; PCR, Polymerase Chain Reaction; ARTP, Atmospheric and Room Temperature Plasma; NCBI, National Center for Biotechnology Information; PPP, Pentose Phosphate Pathway.
