# Supplementary material for: Enhanced production of l-histidine in Escherichia coli through systematic metabolic engineering and CER controlled fermentation
Source: Synth Syst Biotechnol. 2026 May 25;14:333–41. doi: 10.1016/j.synbio.2026.04.038 (PMC13226134; doi:10.1016/j.synbio.2026.04.038)
Supplement: Multimedia component 1 [file mmc1.docx]

**Supplementary Information**

**Enhanced production of L-histidine in *Escherichia coli* through systematic metabolic engineering and CER controlled fermentation**

**Supplementary Methods**

**Effect of exogenous L-histidine supplementation on strains expressing different HisG variants.**

Strains ZL1-1, ZL1-2, and ZL1-3 were cultivated under the same shake-flask fermentation conditions used for the comparison of HisG variants in the main text (Section 2.5). To evaluate their responses to feedback pressure at the cellular level, filter-sterilized L-histidine was added to the fermentation medium at final concentrations of 0, 1, 2, or 5 mM before inoculation. After cultivation, cell growth was determined by measuring OD₆₀₀, and L-histidine in the culture supernatant was quantified using HPLC. All experiments were performed in biological triplicate.

**Quantification of intracellular and extracellular L-histidine.**

Strains ZL14 and ZL15-2 were cultivated under the same shake-flask fermentation conditions used for transporter evaluation. Samples were collected at the end of fermentation for determination of intracellular and extracellular L-histidine concentrations.

For extracellular L-histidine measurement, culture samples were centrifuged at 12,000 × g for 5 min at 4 °C, and the supernatants were filtered through 0.22 μm syringe filters prior to analysis.

For intracellular L-histidine quantification, cell pellets were collected by centrifugation and washed twice with ice-cold PBS buffer to remove residual extracellular metabolites. The washed cells were resuspended in 80% (v/v) methanol and subjected to ultrasonic disruption (3 s on / 3 s off, total 5 min) in an ice bath to extract intracellular metabolites. The lysates were centrifuged at 12,000 × g for 10 min at 4 °C, and the supernatants were collected for L-histidine analysis. Cell dry weight (DCW) was determined by collecting a known volume of culture, washing the cells with distilled water, and drying the biomass at 75 °C until constant weight. Intracellular L-histidine concentrations were normalized to DCW and expressed as mg g⁻¹ DCW.

Both intracellular and extracellular L-histidine concentrations were quantified using high-performance liquid chromatography (HPLC) as described above. All experiments were performed in biological triplicate.

**Structural modeling and comparative analysis of HisG variants**

The three-dimensional structures of HisG_smar_ and HisG*_smar_ were predicted using AlphaFold3. The predicted structures were visualized and analyzed using PyMOL (version 2.5). To identify putative residues involved in L-histidine-mediated allosteric inhibition, the predicted structures were compared with the crystal structure of ATP phosphoribosyltransferase from Mycobacterium tuberculosis (*Mt*ATP-PRT; PDB ID: 1NH7), in which the allosteric histidine-binding site has been reported. Residues corresponding to the allosteric site were identified by structural alignment, and the spatial relationships between these residues and the putative histidine-binding region were examined using PyMOL.

**Supplementary Figure**

**
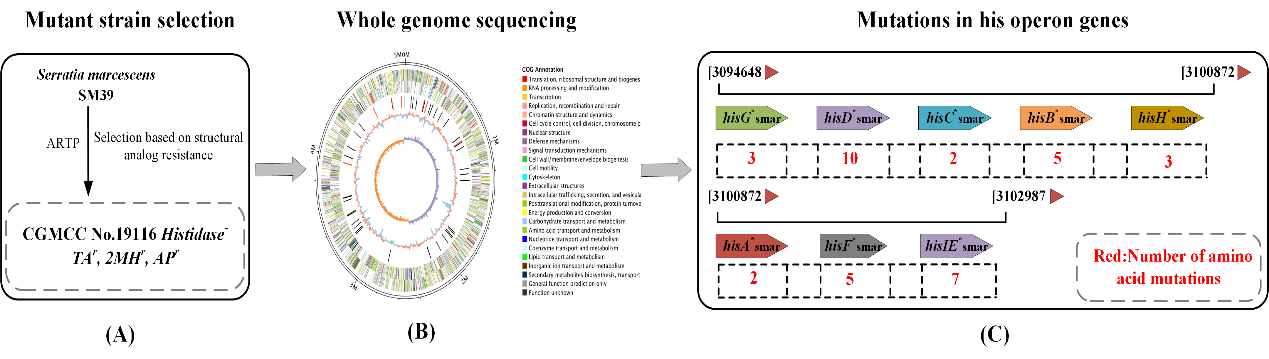
**

**Supplementary Figure 1.** Workflow for beneficial target gene identification in L-histidine production. (A) Source of the high L-histidine-producing mutant strain *S. marcescens* CGMCC No.19116 used in this study. (B) Circular plot of the whole genome sequencing of *S. marcescens* CGMCC No.19116. (C) Overview of mutations in the L-histidine operon genes.


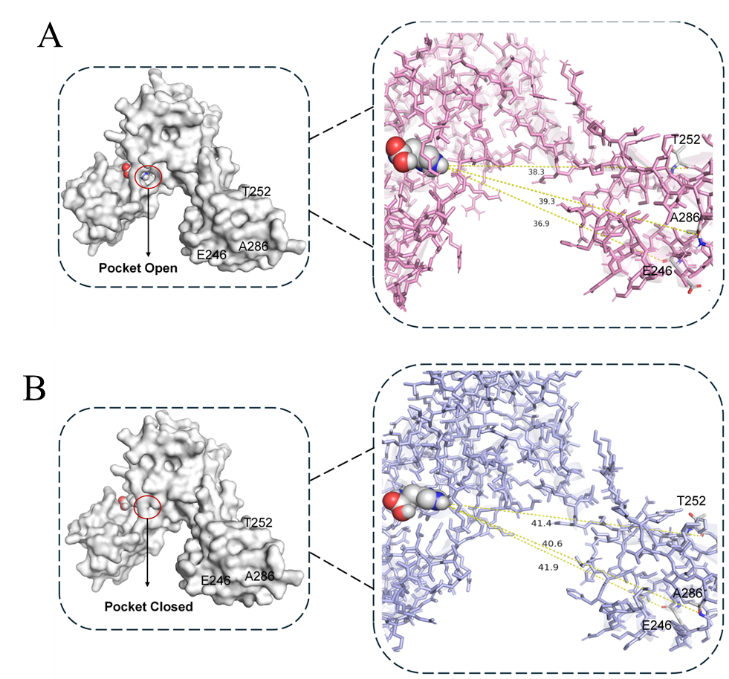


**Supplementary Figure 2** Spatial position of the putative histidine-binding pocket in the predicted structures of HisG_smar_ and HisG*_smar_.

(A) Predicted three-dimensional structure of HisG_smar_ showing the putative histidine-binding region. The protein is shown in outline representation to facilitate visualization of the pocket position, and yellow lines indicate the distances between the putative ligand position and surrounding residues.

(B) Predicted three-dimensional structure of HisG*_smar_ showing the putative histidine-binding region. The protein is shown in outline representation to facilitate visualization of the pocket position, and yellow lines indicate the distances between the putative ligand position and surrounding residues.

**Supplementary Figure 3** Quantification of extracellular and intracellular L-histidine in ZL14 and ZL15-2. Extracellular L-histidine concentrations in the culture supernatants and intracellular L-histidine concentrations normalized to cell dry weight (DCW) were measured after shake-flask fermentation. Data represent mean ± SD of three independent experiments.


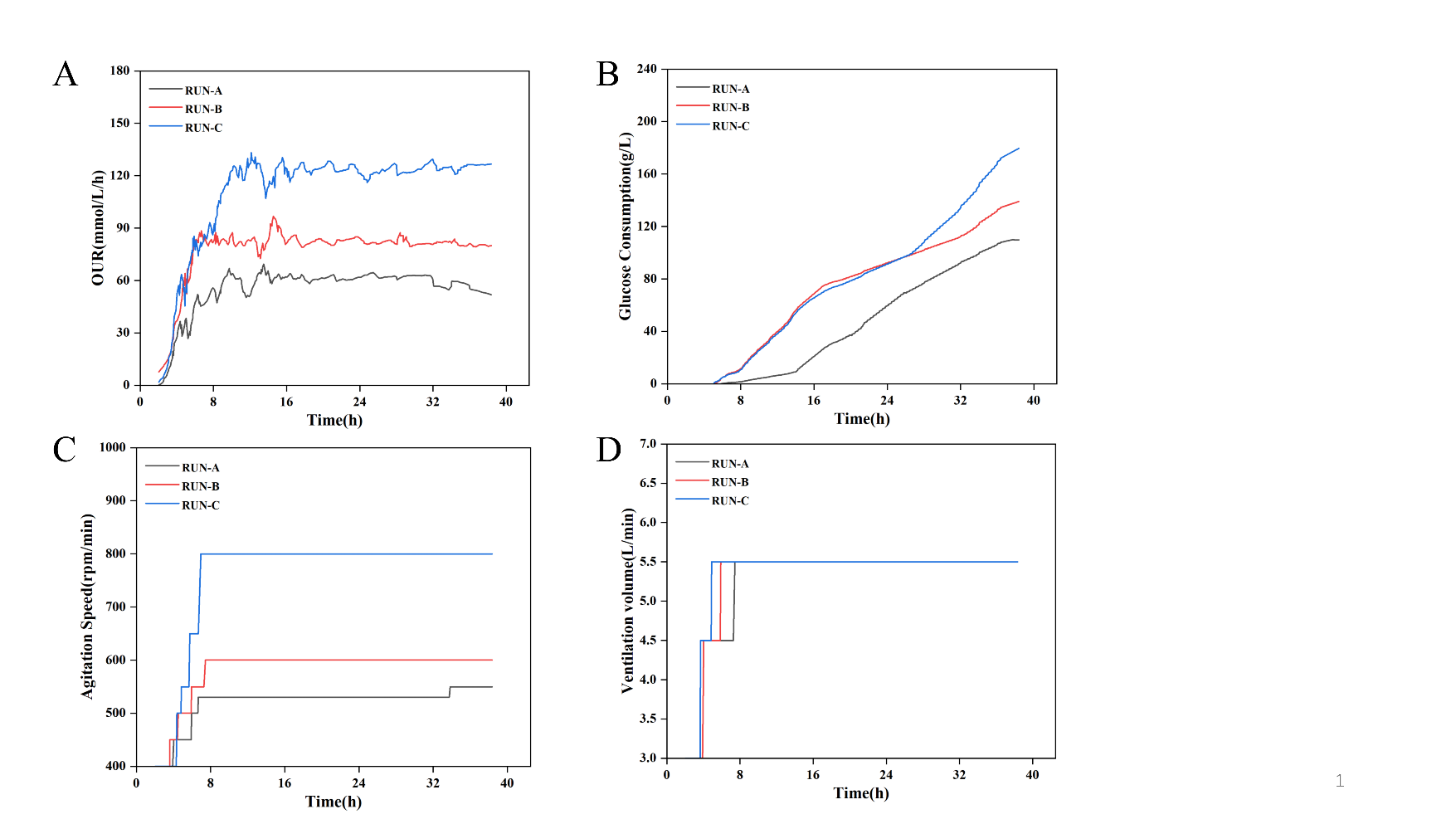


**Supplementary Figure 4** Time-course profiles of key operational parameters during CER-controlled fed-batch fermentation. (A) Oxygen uptake rate (OUR); (B) Glucose feeding amount; (C) Agitation speed; (D) Aeration rate. The profiles correspond to fermentations operated at three CER setpoints. Run A was conducted at 60 mmol L⁻¹ h⁻¹, Run B at 80 mmol L⁻¹ h⁻¹, and Run C at 120 mmol L⁻¹ h⁻¹.

**Supplementary Table 1**  Strains and plasmids used in this study.

| Strains/Plasmids | Characteristics | Sources |
| --- | --- | --- |
| Strains  *E. coli* DH5α  *E. coli* MG1655  *C. glutamicum 13032*  *B. subtilis 168*  *S. marcescens* SM39  *S. marcescens*  CGMCC No.19116 | Host for cloning  Wild type, donor of *leuE,* gene starting strain  wild type, donor of *cgl2458*, *cgl1374*, *lysE* genes  wild type, donor of *rocG* gene  wild type, donor of *hisG* gene  High L-histidine-producing mutant strain previously obtained in this laboratory through ARTP mutagenesis and screening; donor of the mutant L-histidine operon genes | this lab  this lab  this lab  this lab  this lab  this lab |
| ZL1-1  ZL1-2  ZL1-3 | *yciQ*::P*_trc_*-*hisG*_smar_  *yciQ*::P*_trc_*-*hisG**_smar_  *yciQ*::P*_trc_*-*hisG*_cgl_^S143F^ | This study  This study  This study |
| ZL2 | ZL1-2, *△purR* | This study |
| ZL3 | ZL2, *yghX*::P*_trc_*-*prs* | This study |
| ZL4 | ZL3, *yeeP*::P*_trc_*-*zwf* | This study |
| ZL5 | ZL4, *rph*::P*_trc_*-*gnd* | This study |
| ZL6 | ZL5, *mbhA*::P*_trc_*-*hisD**_smar_ | This study |
| ZL7 | ZL6, *yjiV*::P*_trc_*-*hisB**_smar_ | This study |
| ZL8 | ZL7, *gapC*::P*_trc_*-*hisC**_smar_ | This study |
| ZL9 | ZL8, *yeeL*::P*_trc_*-*hisA**_smar_ | This study |
| ZL10 | ZL9, *ygaY*::P*_trc_*-*hisF/H**_smar_ | This study |
| ZL11 | ZL10, *yjgX*::P*_trc_*-*hisIE**_smar_ | This study |
| ZL12  ZL13  ZL14 | ZL11, *ycgH*::P*_trc_*-*rocG*  ZL12, *yjiP*::P*_trc_*-*pntAB*  ZL13, *fhiA*::P*_trc_*-*sthA* | This study  This study  This study |
| ZL15-1 | ZL14, *yghE*::P*_trc_*-*cgl2458* | This study |
| ZL15-2 | ZL14, *yghE*::P*_trc_*-*cgl1374* | This study |
| ZL15-3 | ZL14, *yghE*::P*_trc_*-*leuE* | This study |
| ZL15-4 | ZL14, *yghE*::P*_trc_*-*lysE* | This study |
| ZL16-1 | ZL15-2, *△pgi* | This study |
| ZL16-2 | ZL15-2, P*_pgi_*::P*_rpsL_* | This study |
| ZL16-3 | ZL15-2, P*_pgi_*::P*_rpsT_* _P1_ | This study |
| ZL16-4  Plasmids  pREDCas9  pGRB | ZL15-2, P*_pgi_*::P*_rrnC_* _P1_  Sper, Cas9 and λ Red recombinase  expression vector  Ampr, gRNA expression vector | This study  [23]  [23] |

**Supplementary Table 2.** The primers for cloning works used in this study.

| Primer names | Sequences(5’-3’) |
| --- | --- |
| UP-*yciQ*-S | TTACTTGAAGCATTGGGCGAAC |
| UP-*yciQ*-A | AATTGTTATCCGCTCACAATTCCACACATTATACGAGCCGGATGATTAATTGTCAACCAGTCAAGATGCCAGGGTTCA |
| *hisG*_smar_*-S | TCCGGCTCGTATAATGTGTGGAATTGTGAGCGGATAACAATTTCACACAGGAAACAGACCATGCTGGACAAGACACGTTTACG |
| *hisG*_smar_*-A | CACCGACAAACAACAGATAAAACGAAAGGCCCAGTCTTTCGACTGAGCCTTTCGTTTTATTTGTTACTCCATCATCTTTTCAATCGG |
| DN-*yciQ*-S | AAAGACTGGGCCTTTCGTTTTATCTGTTGTTTGTCGGTGAACGCTCTCCTGAGTAGGACAAATCAGCAAATCCTGATTCGTGTTA |
| DN-*yciQ*-A | TTAACGCAAGGCTTGTCATCAT |
| UP-*△purR*-S | GTCAAACCCACGGCTACGA |
| UP-*△purR*-A | TTAACGACGATAGTCGCGGAACGCTACATCTTTTATTGTTGCCAT |
| DN-*△purR*-S | ATGGCAACAATAAAAGATGTAGCGTTCCGCGACTATCGTCGTTAA |
| DN-*△purR*-A | TTGTTGCCAGTCACCACCC |
| UP-*yghx*-S | TTGGTAGAGATAATCAGTTCATCGC |
| UP-*yghx*-A | AATTGTTATCCGCTCACAATTCCACACATTATACGAGCCGGATGATTAATTGTCAAAGTAATCCAGCAACTCTTGTGGG |
| *prs*-S | TCCGGCTCGTATAATGTGTGGAATTGTGAGCGGATAACAATTTCACACAGGAAACAGACCATGCCTGATATGAAGCTTTTTGC |
| *prs*-A | CACCGACAAACAACAGATAAAACGAAAGGCCCAGTCTTTCGACTGAGCCTTTCGTTTTATTTGTTAGTGTTCGAACATGGCAGAGAT |
| DN-*yghx*-S | AAAGACTGGGCCTTTCGTTTTATCTGTTGTTTGTCGGTGAACGCTCTCCTGAGTAGGACAAATTAGGTTTATCTCTTACGGGATTACGTC |
| DN-*yghx*-A | TACACTAATGCTTCTTCCCTTCGTT |
| UP-*yeep*-S | GGTCAGGAGGTAACTTATCAGCG |
| UP-*yeep*-A | AATTGTTATCCGCTCACAATTCCACACATTATACGAGCCGGATGATTAATTGTCAAATGGCAGGGCTCCGTTTT |
| *zwf*-S | TCCGGCTCGTATAATGTGTGGAATTGTGAGCGGATAACAATTTCACACAGGAAACAGACCATGGCGGTAACGCAAACAGC |
| *zwf*-A | CACCGACAAACAACAGATAAAACGAAAGGCCCAGTCTTTCGACTGAGCCTTTCGTTTTATTTGTTACTCAAACTCATTCCAGGAACG |
| DN-*yeep*-S | AAAGACTGGGCCTTTCGTTTTATCTGTTGTTTGTCGGTGAACGCTCTCCTGAGTAGGACAAATGAACTGGATTTTCTTCTGAACCTGT |
| DN-*yeep*-A | ACGATGTCAGCAGCCAGCA |
| UP-*rph*-S | GTTGATTTCCCCTTCGTCACTCT |
| UP-*rph*-A | AATTGTTATCCGCTCACAATTCCACACATTATACGAGCCGGATGATTAATTGTCAAAAACAAATGTGGCTGCGCAT |
| *gnd*-S | TCCGGCTCGTATAATGTGTGGAATTGTGAGCGGATAACAATTTCACACAGGAAACAGACCATGTCCAAGCAACAGATCGGC |
| *gnd*-A | CACCGACAAACAACAGATAAAACGAAAGGCCCAGTCTTTCGACTGAGCCTTTCGTTTTATTTGTTAATCCAGCCATTCGGTATGG |
| DN-*rph*-S | AAAGACTGGGCCTTTCGTTTTATCTGTTGTTTGTCGGTGAACGCTCTCCTGAGTAGGACAAATGGTTTGCGATCTGGAATACGT |
| DN-*rph*-A | TCGCTCCTCATCTTACTTTTC |
| UP-*mbhA*-S | GCCAGCACGAACATAATCCC |
| UP-*mbhA*-A | AATTGTTATCCGCTCACAATTCCACACATTATACGAGCCGGATGATTAATTGTCAACGAGCGTATTTCCGAGGTTT |
| *hisD*_smar_* -S | TCCGGCTCGTATAATGTGTGGAATTGTGAGCGGATAACAATTTCACACAGGAAACAGACCATGTCGACCTTCAACACGCC |
| *hisD*_smar_*-A | AGTTGCTGGATTACTATGACCCTAGAAGAAATCAACCAGCGCATCAGAAAGTCTCCTGTGCATTCATGCTTGCTCCTTCAGGG |
| DN-*mbhA*-S | AAAGACTGGGCCTTTCGTTTTATCTGTTGTTTGTCGGTGAACGCTCTCCTGAGTAGGACAAATGACCAAAAGTGCGTCCGATAC |
| DN-*mbhA*-A | CGGCGTAATCACAAACTGGC |
| UP-*yjiv*-S | GACTGTGGAAGCCCTGTATACG |
| UP-*yjiv*-A | AATTGTTATCCGCTCACAATTCCACACATTATACGAGCCGGATGATTAATTGTCAATAATCAACGGAGGCTGAAAACT |
| *hisB*_smar_*-S | TCCGGCTCGTATAATGTGTGGAATTGTGAGCGGATAACAATTTCACACAGGAAACAGACCGTGAGCCAAAAAATCCTCTTTATCG |
| *hisB*_smar_*-A | CACCGACAAACAACAGATAAAACGAAAGGCCCAGTCTTTCGACTGAGCCTTTCGTTTTATTTGTCACAGCACTCCTTTCGAGCT |
| DN-*yjiv*-S | AAAGACTGGGCCTTTCGTTTTATCTGTTGTTTGTCGGTGAACGCTCTCCTGAGTAGGACAAATGAGTGGCACCTGAATGACGAA |
| DN-*yjiv*-A | AATCCTGAAAATCCGCTATGCT |
| UP-*gapC*-S | TGGGAAGAAACCACGAAACTC |
| UP-*gapC*-A | AATTGTTATCCGCTCACAATTCCACACATTATACGAGCCGGATGATTAATTGTCAATGTTTCAGCAGGTAGGCGAGA |
| *hisC*_smar_*-S | TCCGGCTCGTATAATGTGTGGAATTGTGAGCGGATAACAATTTCACACAGGAAACAGACCATGAGCATCGAAAAACTGGCG |
| *hisC*_smar_*-A | CACCGACAAACAACAGATAAAACGAAAGGCCCAGTCTTTCGACTGAGCCTTTCGTTTTATTTGTCACATTGGCTCCTGGCG |
| DN-*gapC*-S | AAAGACTGGGCCTTTCGTTTTATCTGTTGTTTGTCGGTGAACGCTCTCCTGAGTAGGACAAATAAAACGGTCGCCTGGTACG |
| DN-*gapC*-A | TTATCCGCCGACATTGCTG |
| UP-*yeel*-S | TTCATCGGGACGAGTGGAGA |
| UP-*yeel*-A | AATTGTTATCCGCTCACAATTCCACACATTATACGAGCCGGATGATTAATTGTCAACCATAGCATCGCCAATCTGA |
| UP-*yjiv*-S | GACTGTGGAAGCCCTGTATACG |
| UP-*yjiv*-A | AATTGTTATCCGCTCACAATTCCACACATTATACGAGCCGGATGATTAATTGTCAATAATCAACGGAGGCTGAAAACT |
| DN-*yjiv*-S | AAAGACTGGGCCTTTCGTTTTATCTGTTGTTTGTCGGTGAACGCTCTCCTGAGTAGGACAAATGAGTGGCACCTGAATGACGAA |
| DN-*yjiv*-A | AATCCTGAAAATCCGCTATGCT |
| UP-*gapC*-S | TGGGAAGAAACCACGAAACTC |
| UP-*gapC*-A | AATTGTTATCCGCTCACAATTCCACACATTATACGAGCCGGATGATTAATTGTCAATGTTTCAGCAGGTAGGCGAGA |
| DN-*gapC*-S | AAAGACTGGGCCTTTCGTTTTATCTGTTGTTTGTCGGTGAACGCTCTCCTGAGTAGGACAAATAAAACGGTCGCCTGGTACG |
| DN-*gapC*-A | TTATCCGCCGACATTGCTG |
| UP-*yeel*-S | TTCATCGGGACGAGTGGAGA |
| UP-*yeel*-A | AATTGTTATCCGCTCACAATTCCACACATTATACGAGCCGGATGATTAATTGTCAACCATAGCATCGCCAATCTGA |
| *hisA*_smar_*-S | TCCGGCTCGTATAATGTGTGGAATTGTGAGCGGATAACAATTTCACACAGGAAACAGACCATGATTATTCCGGCTTTGGATTT |
| *hisA*_smar_*-A | CACCGACAAACAACAGATAAAACGAAAGGCCCAGTCTTTCGACTGAGCCTTTCGTTTTATTTGTTATCCGTTTTGCCAGCATGC |
| DN-*yeel*-S | AAAGACTGGGCCTTTCGTTTTATCTGTTGTTTGTCGGTGAACGCTCTCCTGAGTAGGACAAATACCCAAAGGTGAAGATAAAGCC |
| DN-*yeel*-A | CATTCCCTCTACAGAACTAGCCCT |
| UP-*ygay*-S | GGAGGTGATTGATATTCCGTTCT |
| UP-*ygay*-A | AATTGTTATCCGCTCACAATTCCACACATTATACGAGCCGGATGATTAATTGTCAAACACCGAAGCAACCCAAAAG |
| *hisF/H*_smar_*-S | TCCGGCTCGTATAATGTGTGGAATTGTGAGCGGATAACAATTTCACACAGGAAACAGACCATGCTGGCAAAACGGATAATCC |
| *hisF/H*_smar_*-A | CACCGACAAACAACAGATAAAACGAAAGGCCCAGTCTTTCGACTGAGCCTTTCGTTTTATTTGCTACATCTCCAGAAAGTTTTTCAACAG |
| DN-*ygay*-S | AAAGACTGGGCCTTTCGTTTTATCTGTTGTTTGTCGGTGAACGCTCTCCTGAGTAGGACAAATTTGCTTGCCGCTCCACC |
| DN-*ygay*-A | GGAGTAGGGCTTTCCATAGAGTGT |
| UP-*yjgx*-S | GGAAGTCAACGGGTTATGCG |
| UP-*yjgx*-A | AATTGTTATCCGCTCACAATTCCACACATTATACGAGCCGGATGATTAATTGTCAAGCCTTCGCTCCTCTGACATTAC |
| *hisIE*_smar_*-S | TCCGGCTCGTATAATGTGTGGAATTGTGAGCGGATAACAATTTCACACAGGAAACAGACCGTGCTGACAGAACAACAGAGAAACC |
| *hisIE*_smar_-*A | CACCGACAAACAACAGATAAAACGAAAGGCCCAGTCTTTCGACTGAGCCTTTCGTTTTATTTGTCACGCTTTTTTCTGATGCCG |
| DN-*yjgx*-S | AAAGACTGGGCCTTTCGTTTTATCTGTTGTTTGTCGGTGAACGCTCTCCTGAGTAGGACAAATACAGTGTCTTCCCTGAGCCG |
| DN-*yjgx*-A | GGCGAAGGATACCATCAAGC |
| UP-*ycgh*-S | AATTCAGTCGAGTCGGGTGG |
| UP-*ycgh*-A | AATTGTTATCCGCTCACAATTCCACACATTATACGAGCCGGATGATTAATTGTCAATTGTGCCGCTGACGAGTTTA |
| *rocG*-S | TCCGGCTCGTATAATGTGTGGAATTGTGAGCGGATAACAATTTCACACAGGAAACAGACCATGTCAGCAAAGCAAGTCTCGA |
| *rocG*-A | CACCGACAAACAACAGATAAAACGAAAGGCCCAGTCTTTCGACTGAGCCTTTCGTTTTATTTGTTAGACCCATCCGCGGAAA |
| DN-*ycgh*-S | AAAGACTGGGCCTTTCGTTTTATCTGTTGTTTGTCGGTGAACGCTCTCCTGAGTAGGACAAATGCCCTGAATAAATCCTTTGGTCT |
| DN-*ycgh*-A | GACCCGCAAATGCTGTTGAT |
| Up-*fhiA*-S | GGGCAATGGTGTTGATACTGG |
| Up-*fhiA*-A | AATTGTTATCCGCTCACAATTCCACACATTATACGAGCCGGATGATTAATTGTCAAATCGCCAGAATCATCATCCC |
| *pntAB*-S | TCCGGCTCGTATAATGTGTGGAATTGTGAGCGGATAACAATTTCACACAGGAAACAGACCATGCGAATTGGCATACCAAGA |
| *pntAB*-A | CACCGACAAACAACAGATAAAACGAAAGGCCCAGTCTTTCGACTGAGCCTTTCGTTTTATTTGTTACAGAGCTTTCAGGATTGCATC |
| DN-*fhiA*-S | AAAGACTGGGCCTTTCGTTTTATCTGTTGTTTGTCGGTGAACGCTCTCCTGAGTAGGACAAATCAAGCAGGAGCTGACGGTGT |
| DN-*fhiA*-A | TGCACCAATGCTGGATACTTACA |
| UP-*ylbE*-S  UP-*ylbE*-A | ACCCAACCTTACGCAACCAG  AATTGTTATCCGCTCACAATTCCACACATTATACGAGCCGGATGATTAATTGTCAATTGTTCGATAACCGCAGCAT |
| *sthA*-S | TCCGGCTCGTATAATGTGTGGAATTGTGAGCGGATAACAATTTCACACAGGAAACAGACCATGCCACATTCCTACGATTACGA |
| *sthA*-A | CACCGACAAACAACAGATAAAACGAAAGGCCCAGTCTTTCGACTGAGCCTTTCGTTTTATTTGTTAAAACAGGCGGTTTAAACCG |
| DN-*ylbE*-S | AAAGACTGGGCCTTTCGTTTTATCTGTTGTTTGTCGGTGAACGCTCTCCTGAGTAGGACAAATCGCTGGCGTGCTTTGAA |
| DN-*ylbE*-A | GGCGTAACTCAGCAGGCAG |
| UP-*yghE*-S | GTCAGGCACTGGCGAAAGAT |
| UP-*yghE-*A | AATTGTTATCCGCTCACAATTCCACACATTATACGAGCCGGATGATTAATTGTCAACGCAAGCCATAAACCCACA |
| *Cgl1374*-S | TCCGGCTCGTATAATGTGTGGAATTGTGAGCGGATAACAATTTCACACAGGAAACAGACCATGATCCGCAAACTTGCTCG |
| *Cgl1374*-A | CACCGACAAACAACAGATAAAACGAAAGGCCCAGTCTTTCGACTGAGCCTTTCGTTTTATTTGTTAAAGTTCGATGCCCTTCAGC |
| DN-*yghE-*S | AAAGACTGGGCCTTTCGTTTTATCTGTTGTTTGTCGGTGAACGCTCTCCTGAGTAGGACAAATTTCCGACATCGAAATGCGT |
| DN-*yghE-*A | AGGCGTTGTTGTGGCAGATT |
| UP-P*_pgi_*-P*_rrnC_* _P1_-S | CCTTCATTGAGACGTGGGAGC |
| UP-P*_pgi_*-P*_rrnC_* _P1_-A | TTTCTGACCGCGCATTTTTTCAGCAAAAGCCCCGTTAATGTGCCATAAAACAAGCAATTTGCCGTTTTTGCAACCGTAATCACACTTCC |
| DN-P*_pgi_*-P*_rrnC_* _P1_-S | GCGCGGTCAGAAAATTATTTTAAATTTCCTCTTGTCAGGCCGGAATAACTCCCTATAATGCGCCACCAATGAAAAACATCAATCCAAcGC |
| DN-P*_pgi_*-P*_rrnC_* _P1_-A | TGCTTTGCCGGTATAACCTTTC |

**Supplementary Table 3.** The growth phase-dependent promoters used to control the expression of *pgi* gene.

| Denotation | Promoter sequences |
| --- | --- |
| rpsL | TCGTCAGACTTACGGTTAAGCACCCCAGCCAGATGGCCTGGT  GATGGCGGGATCGTTGTATATTTCTTGACACCTTTTCGGCATC  GCCCTAAAATTCGGCGTCCTCATA |
| rpsT P1 | TCATTGCCATGGCGCAAATCACGGGAAGAAACTGACCGCCTG  CTGCAATTTTTATCGCGGAAAAGCTGTATTCACACCCCGCAAG  CTGGTAGAATCCTGCGCCATCACTACGTAACGAGT |
| rrnC P1 | CTTAAAGGCATTACTTATCTTCCTTTTTCTTTTTATTCCTCCTTAG  TATGCCACCAGGAAGTGTGATTACGGTTGCAAAAACGGCAAA  TTGCTTGTTTTATGGCACATTAACGGGGCTTTTGCTGAAAAAAT  GCGCGGTCAGAAAATTATTTTAAATTTCCTCTTGTCAGGCCGG  AATAACTCCCTATAATGCGCCACCA |

**Supplementary Table 4** Effects of Different Carbon Dioxide Release Rate Controls on Histidine Fermentation

| CER(mmol/L/h) | 60±3.8 | 80±2.5 | 120±6.9 |
| --- | --- | --- | --- |
| Bacterial concentration(g/L) | 33±1.5 | 36.4±2.1 | 38.4±1.9 |
| L-histidine(g/L) | 32.2±2.1 | 49.8±1.9 | 42.8±2.2 |
| Conversion efficiency(%) | 25.7±1.2 | 26.5±1.1 | 24.5±0.8 |
| Acetic acid(g/L) | 4.2±0.4 | 0.5±0.1 | 0.2±0.1 |

**Supplementary Table S5** Effects of exogenous L-histidine supplementation on cell growth and L-histidine production of strains expressing different HisG variants.

| Exogenous L-histidine (mM) | Strain | Net L-histidine titer (g/L) | Final OD_600_ | Relative titer retention (%) |
| --- | --- | --- | --- | --- |
| 0 | ZL1-1 | 0.145 ± 0.008 | 49.5 ± 1.2 | 100 |
| 1 | ZL1-1 | 0.136 ± 0.007 | 48.8 ± 1.1 | 93.8 |
| 2 | ZL1-1 | 0.125 ± 0.006 | 47.0 ± 1.0 | 86.2 |
| 5 | ZL1-1 | 0.080 ± 0.005 | 41.5 ± 0.9 | 55.2 |
| 0 | ZL1-2 | 0.350 ± 0.012 | 42.5 ± 1.0 | 100 |
| 1 | ZL1-2 | 0.346 ± 0.011 | 42.1 ± 1.0 | 98.9 |
| 2 | ZL1-2 | 0.335 ± 0.010 | 41.0 ± 0.9 | 95.7 |
| 5 | ZL1-2 | 0.255 ± 0.009 | 38.2 ± 0.8 | 72.9 |
| 0 | ZL1-3 | 0.225 ± 0.010 | 42.0 ± 1.1 | 100 |
| 1 | ZL1-3 | 0.210 ± 0.009 | 41.4 ± 1.0 | 93.3 |
| 2 | ZL1-3 | 0.190 ± 0.009 | 39.8 ± 0.9 | 84.4 |
| 5 | ZL1-3 | 0.135 ± 0.007 | 37.2 ± 0.8 | 60.0 |

*L-histidine titers represent the net L-histidine produced after subtraction of the externally supplemented L-histidine. Relative titer retention (%) was calculated relative to the titer obtained in the absence of exogenous L-histidine.

**Reference**

[23] Li Y, Lin Z, Huang C, Zhang Y, Wang Z, Tang YJ, et al. Metabolic engineering of *Escherichia coli* using CRISPR-Cas9 meditated genome editing. Metab Eng 2015;31:13-21. <https://doi.org/10.1016/j.ymben.2015.06.006>.
